# Thought and the Brain

**Published:** 1892-03

**Authors:** Dexter Davis


### THOUGHT AND THE BRAIN.

### ED. HALL'S JOURNAL OF HEALTH.

The Bible proposition, that "As a man thinketh so is he," is very suggestive of inquiries of an interesting character.

But man is not our present topic ; only a small part of his organism —the brain. And even that must be partitioned off, or departmented, in order to meet a few queries arising in the mind of this layman as to the connecting link between mind and matter.

Matter and mind seem to approach each other very closely in the brain. Experiments have been made of late by touching a dozen different nerves in sensitive brains, and said sensitives have given expression to as many different sensations and thoughts by and in their speech at the time. Now, are the phrenological bumps actual organs of conscious mentality? and, if only touched, responsive with a thrill of activity and delight? like an electric button that rings a bell at your door or ignites a light in your parlor?

Cerebrum and cerebellum, medulla-oblongata and pons varolii, tuber annulare and nerve ganglia of sensation and volition, do any or all these pompous medical terms quite tell us common folks what the connecting link is? Hardly!

If, then, the microscope, and the scalpel, and the trained eye of the anatomist, cannot discern sign or show of remaining intelligence or will power in the brain of a cadaver, can we still believe in the materialism of human mentality? Not very much, and be candid as to our convictions.

But let us shift the point of observation: Can you find any thing in the gray or white brain substance indicative of a purely mental or metaphysical character? Not an atom.

If you could combine the contents of ten ordinary skulls, would you be able to produce the poetical fury of a Byron? the majestic and statesman-like utterances of a Webster? the inimitable mimicry of John B. Gough? or the rich and entertaining drollery of the late Artemus Ward? Of course not, and never.

The brain, with all its solid, liquid and ethereal elements in it, may be weighed in pounds and ounces, and measured in cubic inches, but with what tape line or scales will you measure and weigh Memory, Hope, Logic, Reason, Judgment and Understanding?

Having then considered these few inquiries on the materialistic view, that without Brainal Organism there is no such thing as a Human Spirit, and inferred the absurdity of the proposition that aggregation of brain and nerve atoms determine the quantity and quality of purely mental abilities, making the Poet, the Philosopher or Statesman.

One may ask what is the Brain, and my answer, for want of a better, would have to be: It is as a lens or a prism to the light; as the strings of an æolian harp to the wind that stirs them; as the olfactory nerves to the gases that convey odors; or the gustatory nerve to supply us with a sense of taste. In other words, the Brain is an all-round *sense* organ.

But to catechize consciousness a little further:

Are not thought and mental forces of different individuals so varying and disproportioned to physical character and elemental combinations that we may say that the brain of a genius is simply the seat of a power we call mind; and that the presence of an extraordinary mind or spirit will shape a brain to its liking, and give us the fine contours of the head of a Webster, a Humboldt or a Shakspeare?

Assuming that such is the case, and that spiritual forces mould the matter of the brain, and dispose the tissue in its convolutions, what are the elements that it uses? or, in other words, what is the uniting link between the mind and the brain?

Is it *magnetism*? John Bovee Dods used to say, "It is animal electricity," but he denied the spiritualism and wonderful mediumship of his gifted daughter until his very last days.

The First Napoleon used to say, his "mental faculties were like a chest of drawers or a shelf of books," he "could open and shut, put back one set of mind forces," "and take down another," and "when all were shut up," he "went to sleep."

These contributed queries have arisen in my mind with thousands more on reading the subjoined clip, concerning the post-mortem examination of the brain of Paul Morphy, the champion chess player.

Yours truly,

DEXTER DAVIS.

"Imagine a man concentrating his thought on twelve chess boards, and then on the positions on each board, and then you can conceive what such effort must be. One famous player stated that his plan was this: He formed twelve mental boards. Each had, as it were, a little closet of its own. When the door of number one was closed that of number two was opened, and so on to the last. After one of his games it was always three hours before he could obtain sleep. A remarkable discovery has recently been made with regard to the brain of one of the famous chess players. A young doctor made a post-mortem examination of the cells and arrived at an interesting conclusion. Under a microscope, he says, these cells were exactly the shape of chess boards."

